# RAP3DF - One shoot 3D face dataset

**DOI:** 10.1016/j.dib.2020.106281

**Published:** 2020-09-05

**Authors:** Rafael Alexandre Piemontez, Eros Comunello

**Affiliations:** University of Itajaí Valley – UNIVALI, Santa Catarina, Brazil

**Keywords:** 3D biometrics, 3D faces, Image dataset, Kinect one, RGB, IR, Depth Image

## Abstract

Develop researches in the 3D biometrics field requires a large set of images, whether for training the algorithms or during the recognition test. Several datasets can be found in the literature. In an analysis of these datasets it was observed that a single dataset does not have the types of infrared images, visible and three-dimensional light, for the same sample. Given this context, the present work conceived this 3D facial dataset, with its respective visible light image and infrared spectrum, providing the entire image acquisition process from the Kinect One device. The work consists of 267 samples from 64 volunteers, where each volunteer has a frontal facial image and 3 images in arbitrary positions.

## Specifications Table

**Table:** 

Subject	Computer Science (General)
Specific subject area	Facial biometrics
Type of data	Table, Figure and Files
How data were acquired	Kinect One
Data format	BMP or Kinect Raw
Parameters for data collection	Data Collection:
	• 64 volunteers
	• 267 facial images samples
	• 267 facial images of the visible light spectrum
	• 267 facial images of the infrared spectrum
	• 267 depth images (3D)
	Demographic Information:
	• Age
	• Gender
	• Colour
	• Weight
	• Height
Description of data collection	This dataset contains facial images of volunteers in frontal and arbitrary poses. Each facial image collection has a visible light image, an infrared image and a depth image.
Data source location	The data were collected at different locations in the University of Itajaí Valley – UNIVALI. Santa Catarina - Brazil
Data accessibility	Direct URL to data: http://dx.doi.org/10.17632/kpdkpcs8zb.1
Related research article	

## Value of the Data

• The dataset has infrared facial images, 3D depth images, and images of the visible light spectrum for each sample.
• The data can be used to train classifiers or artificial neural networks to identify a face, age or gender. Different classifiers can be trained to identify the best type of image for a given issue, such as facial recognition.
• Samples from volunteers in random positions and in frontal positions can be used to check whether a facial recognition system can identify faces in different perspectives.
• The dataset can be used to identify the direction of a face from 2d and 3d models / images and also to analyse which one best accurates.

## 1 Data Description

The images in this dataset were collected by a single person during the period of October 10th to 27th of the year 2017. Eight classes from the computer science course at UNIVALI University were invited to participate in this paper, where 64 accepted the invitation.

Two groups of images were extracted for each participating volunteer: in the first group the volunteers established themselves with their faces in the frontal position without performing facial expressions, looking straight at the camera and in the second group of images the volunteers, freely, could look in any direction and perform any facial expression. These facial expressions were classified into 6 different groups: happiness, surprise, fear, sadness, anger and disgust. The nomenclatures of the expression groups are the same groups used in the Bosphorus database [14].

Each sample collected has 3 types of images, a visible light image, an infrared image and a depth image with the 3D representation extracted from Kinect One. All 3 images were collected at the same time. The depth image was created by calculating the distance from each point of the three-dimensional face to a virtually created surface, located in the same three-dimensional position as the Kinect One camera.

BMP-type images were generated for all types of the collected images, allowing interested parties to quickly see the dataset. “Data” extension files were also saved with the information provided by Kinect One, without manipulation. The “data” extension files of the depth images have in their content a list of floating points of 8 bytes. All of the images are 119 pixels wide and 149 pixels high. Table 1 presents the image type for each file type; the file types are differentiated by the suffix of their names.

**Table 1:** File structure.

File name suffix	Extension	Files image type
k1_box_xy	bmp	Visible light
k1_box_xy_ir_view	bmp	Infra-red
k1_box_xyz_depth_view	bmp	Depth image
k1_box_xyz_ir	raw	Infra-red
k1_box_xyz_depth	raw	Depth image

Each participating volunteer received a unique identifier, which became a folder of the same name, grouping the images of that individual. All images collected and stored in their folders were made available at the link http://dx.doi.org/10.17632/kpdkpcs8zb.1. With the files downloaded on your machine: open the folder and file k1_box_xy.bmp to see the visible light image or open the k1_box_xy_ir_view.bmp file to see the picture with the infrared image or open the k1_box_xyz_depth_view.bmp file to view the depth image. These 3 images represent the frontal portrait of the volunteer.

After data collection 71 volunteers participated in this dataset where only 64 volunteers agreed to make their images available, counting 267 samples of frontal facial and random positions images. Seven volunteers, during the acquisition, gave up participating the dataset.

**Fig. 1:**
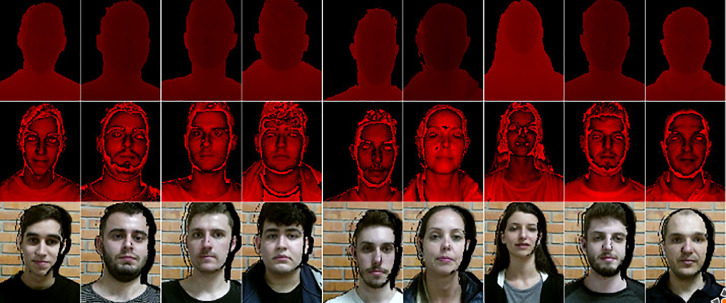
Depth, infrared and visible light images of the participants.

Fig. 1 shows the types of images collected with Kinect One and described below:

• Depth image, with three-dimensional information from the volunteer;
• Infrared image, with information from the infrared spectrum;
• Visible light image, with information on the visible light spectrum.

**Fig. 2:**
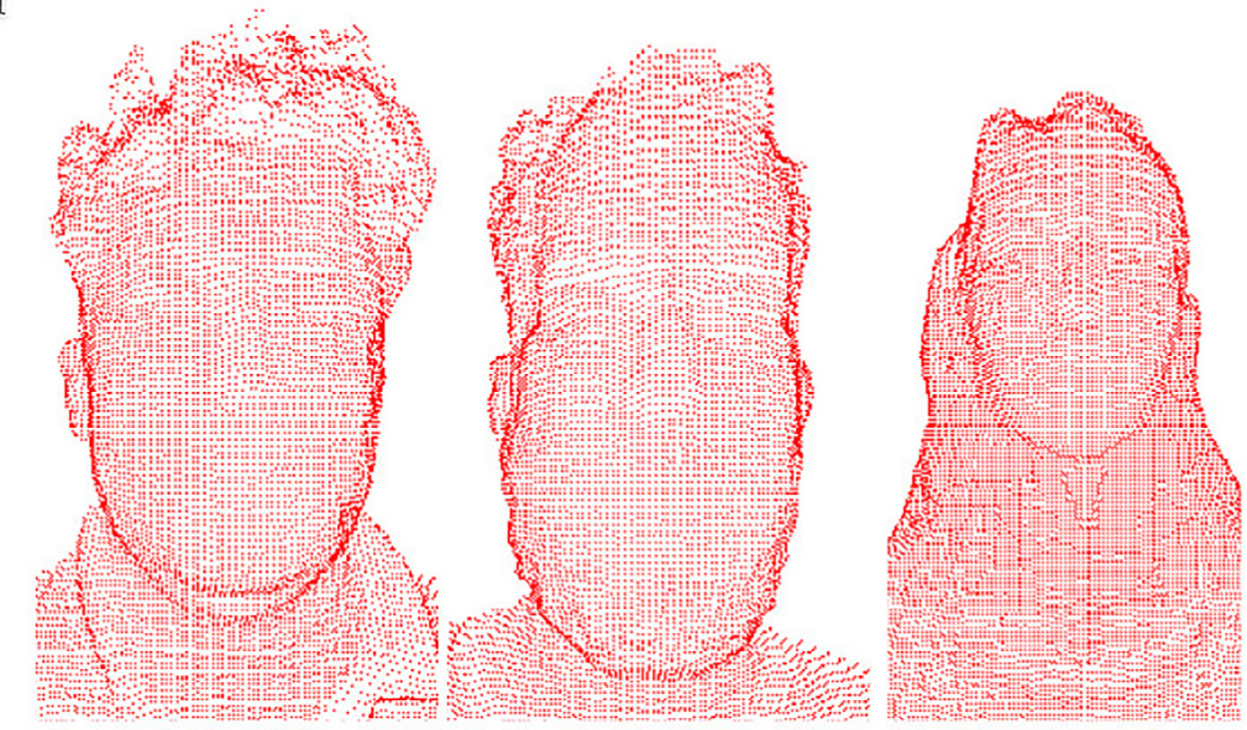
Three-dimensional modelling of the captured faces.

The red colour intensity in the pixels of the depth images represents the distance from a certain point on the face in relation to the Kinect camera at the time of the collection. From this information it is possible to recreate the object three-dimensionally, as shown in Fig. 2.

**Fig. 3:**
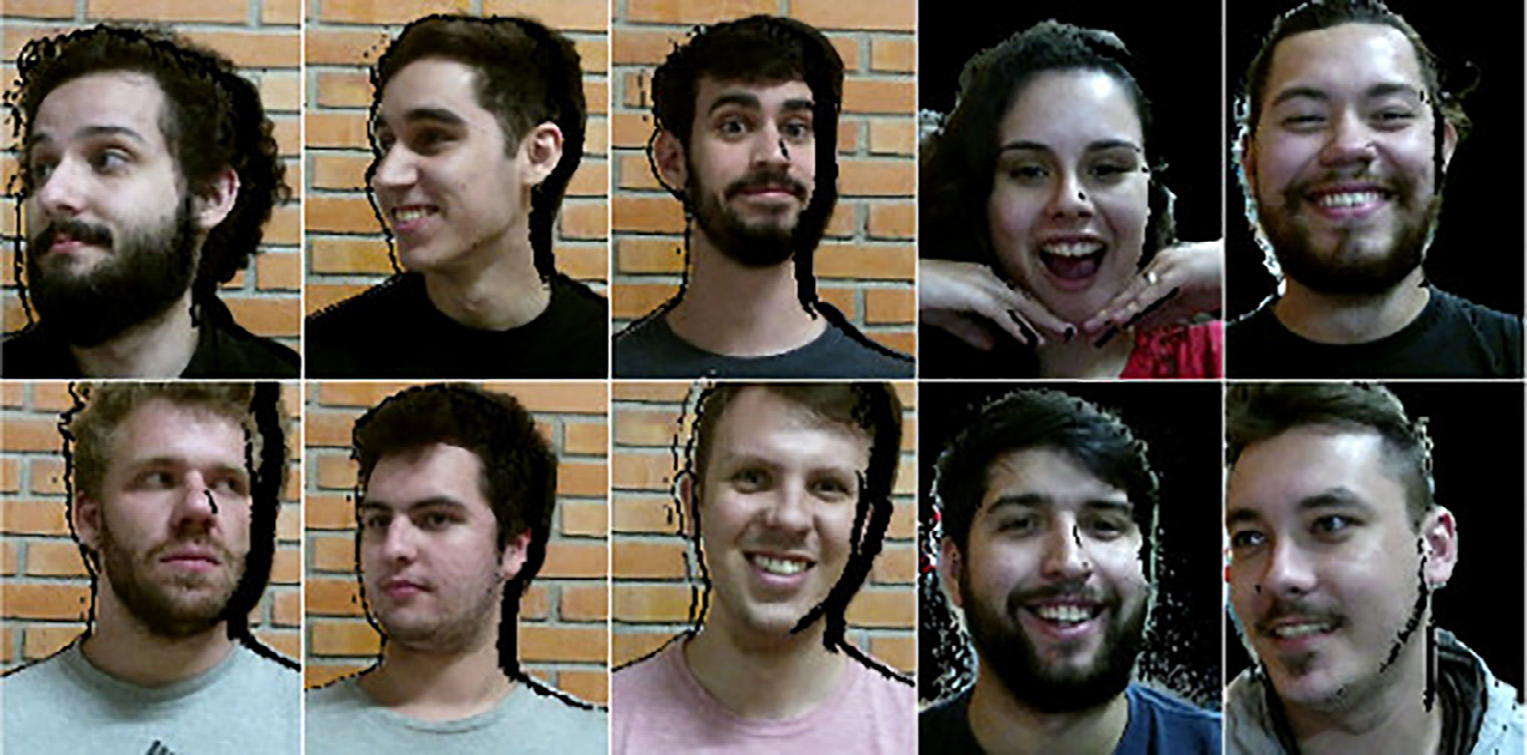
Images of visible light in random positions of the participants.

To allow facial recognition tests to be performed images were also collected in arbitrary positions from the volunteers, who expressed themselves freely in front of the Kinect One camera. Fig. 3 illustrates some collected images. The black background in the volunteers’ facial images occurs due to a configuration created to collect objects less than 2 m away, and also because one of the collection sites did not have a wall behind the volunteers.

**Fig. 4:**
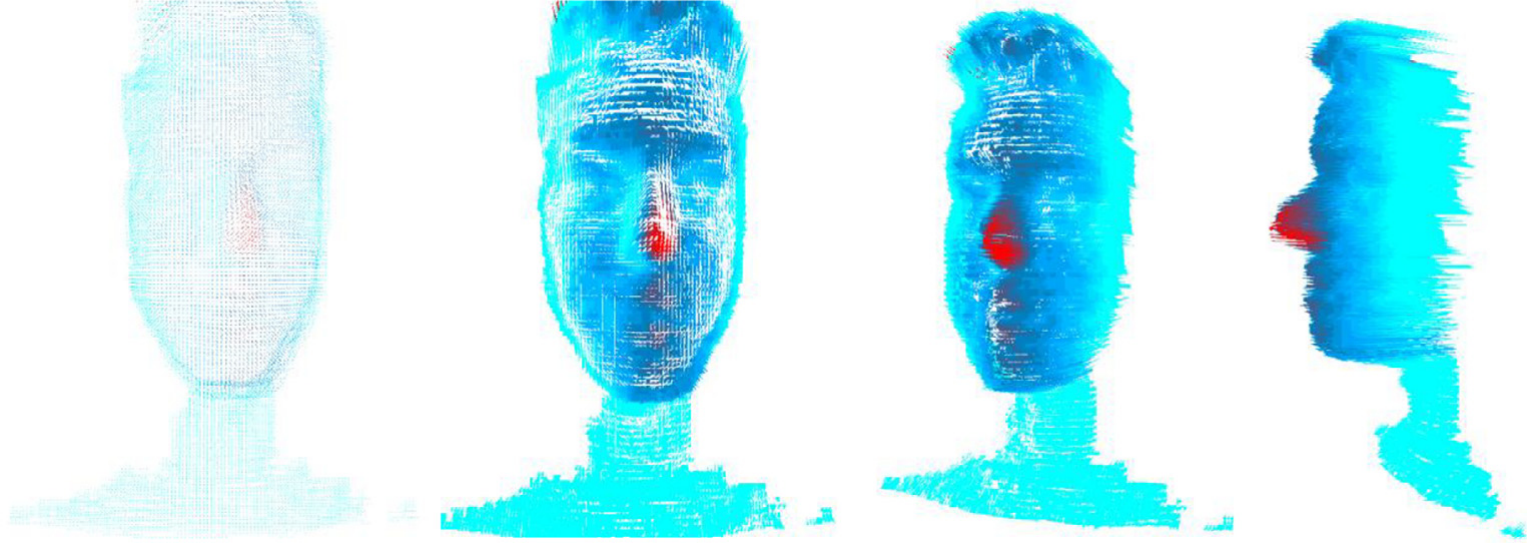
(a) Three-dimensional modelling of points, (b) Three-dimensional triangular modelling, (c) Triangular modelling slightly rotated to the left and (d) Triangular modelling fully rotated to the left.

In Fig. 4b, 4c and 4d we can see the 3D modelling resolution when the triangulation of the points is performed (Fig. 4a). Adjacent pixels were used to triangulate the image.

## 2 Demographic information

For other studies to be developed, with this dataset, demographic information (age, sex, colour, weight and height) was also collected from the participants. This information allows us to perform studies other than facial recognition, such as age and sex detection based on image. Regarding skin colour, the volunteers were instructed to choose as described in [7].

## 3 Comparison between datasets

To assess the quality of this dataset a comparative analysis was carried out with other datasets. On Table 2 below this comparison is presented, where the first column exhibits the name of the dataset, the second column the population (number of individuals who participated the project), the third column the number of samples in the dataset and from the fourth to the seventh column it is informed with an “X” which type of image the dataset has and the symbol “?” demonstrates the dataset information does not indicates what type of image the dataset has, however the illustrative images on its articles indicate it. Depth images were considered as 3D images.

**Table 2:** Analysis of similar facial datasets.

Data base	Population	Samples	Grey	RGB	IR	3D
[1] The 3d mask Attack Database (3DMAD)	17			X		X
[14] Yale Face Database	15	165	X			
[10] YouTube Faces Database	1995			X		
[8] Indian Movie Face database (IMFDB)	100	34,512		X		
[3] The Japanese Female Facial Expression (JAFFE)	10	213	?	?		
3D RMA database	120					X
[2] 3D Morphable Face Models	200			X		X
[12] BU-3DFE (Binghamton University 3D Facial Expression)	100	2500		X		X
[13] BU-4DFE (3D + time): A 3D Dynamic Facial Expression)	101			X		X
[4] Long Distance Heterogeneous Face Database (LDHF-DB)	100			X	X	
[6] Natural Visible and Infrared Facial Expression (USTC—NVIE)	100			X	X	
[9] BJUT-3D-R1	500			X		X
[5] GavabDB	61	427		X		X
[11] ND-2006	888	13,450				X
RAP3DF (this data base)	64	267		X	X	X

We can see on Table 2 that in none of the datasets we can find all types of images . However, if we consider that a grayscale image is a simplified representation or channel reduction of an RGB, IR or 3D image, the dataset on this article includes all types of images.

## 4 Experimental design, materials and methods

**Fig. 5:**
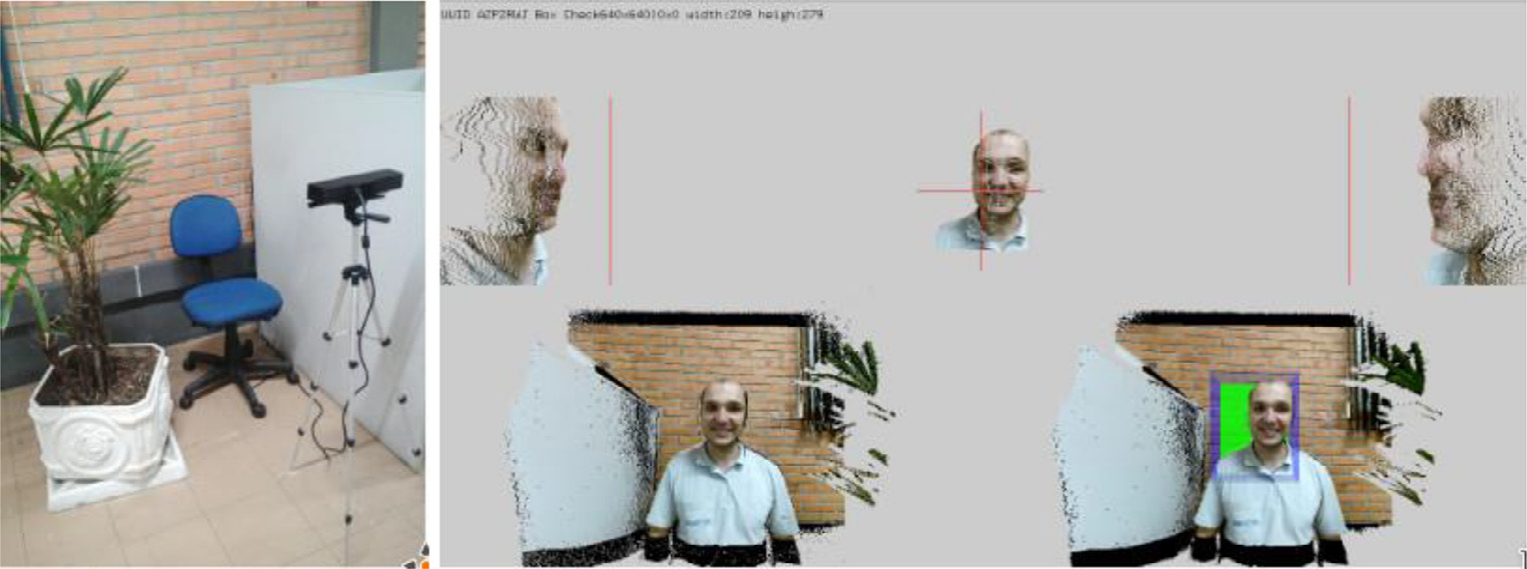
The Environment and the system used for capturing images from Kinect One.

To capture the 3D, infrared and visible light images a structure was established in different locations at UNIVALI - University of Itajaí Valley, so that the volunteers were properly positioned during the capture of the images. For the structure a chair and a tripod with Kinect One, a laptop and 2 monitors were placed, with one monitor for the volunteer to see the images and the second for the researcher to check and collect the images, as shown on Fig. 5a. With the structure assembled a software was used, developed for the purpose of this article, which collects the images from Kinect One to a region of 60 cm in front of the device, extracting all information in an area of 30 cm wide by 30 cm high and 60 cm deep, as shown on Fig. 5b.

## Ethics statement

During the approach and data collection the volunteers were informed about the project, its risks and benefits, the collection procedure, the required participation time, the right to stop participating the dataset without penalties and also all doubts had been solved before data collection began. After clarifications the volunteers were asked if they would like to participate the project, if the response was affirmative they were asked to read and sign a consent form as well as an image use term.

This dataset was created with the approval of CEP (Research Ethics Committee) from University of Itajaí Valley - Brazil with CAAE (Certificate of Presentation of Ethical Appreciation) number: 97615018.9.0000.012.

## Declaration of Competing Interest

None.

## Appendix Supplementary materials

Image, application 1

Image, application 2
