# Supplementary material for: RAP3DF - One shoot 3D face dataset
Source: Data Brief. 2020 Sep 5;32:106281. doi: 10.1016/j.dib.2020.106281 (PMC7509182; doi:10.1016/j.dib.2020.106281)
Supplement: Supplementary file 1 [file mmc1.zip › cas-sc-template.pdf]

Graphical Abstract

This is a specimen a_b title

CV Radhakrishnan,Han Theh Thanh,CV Rajagopal,Rishi T.

Highlights

This is a specimen a_b title

CV Radhakrishnan,Han Theh Thanh,CV Rajagopal,Rishi T.

- Research highlights item 1
- Research highlights item 2
- Research highlights item 3

This is a specimen a_b title[⋆,⋆⋆]

Sir CV Radhakrishnan[a,c,*,1] (Researcher), Han Theh Thanh[b,d], CV Rajagopal Jr[b,c,2] (Co-ordinator) and Rishi T.[a,c,**,1,3]

[a]Elsevier B.V., Radarweg 29, 1043 NX Amsterdam, The Netherlands
[b]Sayahna Foundation, Jagathy, Trivandrum 695014, India
[c]STM Document Engineering Pvt Ltd., Mepukada, Malayinkil, Trivandrum 695571, India

ARTICLE INFO

Keywords:
quadrupole exciton
polariton
WGM
BEC

ABSTRACT

This template helps you to create a properly formatted LaTeX manuscript. `\beginabstract` ...`\endabstract` and `\begin{keyword}` ... `\end{keyword}` which contain the abstract and keywords respectively. Each keyword shall be separated by a `\sep` command.

1. Introduction

The Elsevier cas-sc class is based on the standard article class and supports almost all of the functionality of that class. In addition, it features commands and options to format the

- document style
- baselineskip
- front matter
- keywords and MSC codes
- theorems, definitions and proofs
- lables of enumerations
- citation style and labeling.

This class depends on the following packages for its proper functioning:

1. natbib.sty for citation processing;
2. geometry.sty for margin settings;
3. fleqn.clo for left aligned equations;
4. graphicx.sty for graphics inclusion;
5. hyperref.sty optional packages if hyperlinking is required in the document;

All the above packages are part of any standard LaTeX installation. Therefore, the users need not be bothered about downloading any extra packages.

⋆This document is the results of the research project funded by the National Science Foundation.

⋆⋆The second title footnote which is a longer text matter to fill through the whole text width and overflow into another line in the footnotes area of the first page.

This note has no numbers. In this work we demonstrate a_b the formation Y_1 of a new type of polariton on the interface between a cuprous oxide slab and a polystyrene micro-sphere placed on the slab.

*Corresponding author
**Principal corresponding author
cvr_1@tug.org.in (C. Radhakrishnan); cvr3@sayahna.org (C. Rajagopal); rishi@stmdocs.in (R. T.)
www.cvr.cc, cvr@sayahna.org (C. Radhakrishnan); www.sayahna.org (C. Rajagopal); www.stmdocs.in (R. T.)
ORCID(s): 0000-0001-7511-2910 (C. Radhakrishnan)

[1]This is the first author footnote. but is common to third author as well.

[2]Another author footnote, this is a very long footnote and it should be a really long footnote. But this footnote is not yet sufficiently long enough to make two lines of footnote text.

Figure 1: The evanescent light - $1S$ quadrupole coupling ($g_{1,l}$) scaled to the bulk exciton-photon coupling ($g_{1,2}$). The size parameter kr_0 is denoted as x and the PMS is placed directly on the cuprous oxide sample ($\delta r = 0$, See also Table 1).

2. Installation

The package is available at author resources page at Elsevier (`http://www.elsevier.com/locate/latex`). The class may be moved or copied to a place, usually, `$TEXMF/tex/latex/elsevier/`, or a folder which will be read by LaTeX during document compilation. The TeX file database needs updation after moving/copying class file. Usually, we use commands like `mktexlsr` or `texhash` depending upon the distribution and operating system.

3. Front matter

The author names and affiliations could be formatted in two ways:

(1) Group the authors per affiliation.
(2) Use footnotes to indicate the affiliations.

See the front matter of this document for examples. You are recommended to conform your choice to the journal you are submitting to.

4. Bibliography styles

There are various bibliography styles available. You can select the style of your choice in the preamble of this document. These styles are Elsevier styles based on standard styles like Harvard and Vancouver. Please use BibTeX to generate your bibliography and include DOIs whenever available.

Here are two sample references: See Fortunato (2010). Also refer Fortunato (2010); Newman and Girvan (2004). More citations are here (Fortunato, 2010; Vehlow, Reinhardt and Weiskopf, 2013).

5. Floats

Figures may be included using the command, `\includegraphics` in combination with or without its several options to further control graphic. `\includegraphics` is provided by graphic[s,x].sty which is part of any standard LaTeX distribution. graphicx.sty is loaded by default. LaTeX accepts figures in the postscript format while pdfLaTeX accepts *.pdf, *.mps (metapost), *.jpg and *.png formats. pdfLaTeX does not accept graphic files in the postscript format.

The `table` environment is handy for marking up tabular material. If users want to use multirow.sty, array.sty, etc., to fine control/enhance the tables, they are welcome to load any package of their choice and cas-sc.cls will work in combination with all loaded packages.

Table 1
This is a test caption. This is a test caption. This is a test caption. This is a test caption.

Col 1	Col 2	Col 3	Col4
12345	12345	123	12345
12345	12345	123	12345
12345	12345	123	12345
12345	12345	123	12345
12345	12345	123	12345

6. Theorem and theorem like environments

cas-sc.cls provides a few shortcuts to format theorems and theorem-like environments with ease. In all commands the options that are used with the `\newtheorem` command will work exactly in the same manner. cas-sc.cls provides three commands to format theorem or theorem-like environments:

```
\newtheorem{theorem}{Theorem}
\newtheorem{lemma}[theorem]{Lemma}
\newdefinition{rmk}{Remark}
\newproof{pf}{Proof}
\newproof{pot}{Proof of Theorem \ref{thm2}}
```

The `\newtheorem` command formats a theorem in LaTeX's default style with italicized font, bold font for theorem heading and theorem number at the right hand side of the theorem heading. It also optionally accepts an argument which will be printed as an extra heading in parentheses.

```
\begin{theorem}
 For system (8), consensus can be achieved with
 $\|T_{\omega z}$ ...
   \begin{eqnarray}\label{10}
   ....
   \end{eqnarray}
\end{theorem}
```

Theorem 1. *For system (8), consensus can be achieved with* $\|T_{\omega z}$ ...

$$.... \tag{1}$$

The `\newdefinition` command is the same in all respects as its `\newtheorem` counterpart except that the font shape is roman instead of italic. Both `\newdefinition` and `\newtheorem` commands automatically define counters for the environments defined.

The `\newproof` command defines proof environments with upright font shape. No counters are defined.

7. Enumerated and Itemized Lists

cas-sc.cls provides an extended list processing macros which makes the usage a bit more user friendly than the default LaTeX list macros. With an optional argument to the `\begin{enumerate}` command, you can change the list counter type and its attributes.

```
\begin{enumerate}[1.]
\item The enumerate environment starts with an optional
  argument '1.', so that the item counter will be suffixed
  by a period.
\item You can use 'a)' for alphabetical counter and '(i)' for
  roman counter.
```

```
\begin{enumerate}[a)]
  \item Another level of list with alphabetical counter.
  \item One more item before we start another.
  \item One more item before we start another.
  \item One more item before we start another.
  \item One more item before we start another.
```

Further, the enhanced list environment allows one to prefix a string like 'step' to all the item numbers.

```
\begin{enumerate}[Step 1.]
 \item This is the first step of the example list.
 \item Obviously this is the second step.
 \item The final step to wind up this example.
\end{enumerate}
```

8. Cross-references

In electronic publications, articles may be internally hyperlinked. Hyperlinks are generated from proper cross-references in the article. For example, the words Fig. 1 will never be more than simple text, whereas the proper cross-reference `\ref{tiger}` may be turned into a hyperlink to the figure itself: Fig. 1. In the same way, the words Ref. [1] will fail to turn into a hyperlink; the proper cross-reference is `\cite{Knuth96}`. Cross-referencing is possible in LaTeX for sections, subsections, formulae, figures, tables, and literature references.

9. Bibliography

Two bibliographic style files (`*.bst`) are provided — model1-num-names.bst and model2-names.bst — the first one can be used for the numbered scheme. This can also be used for the numbered with new options of natbib.sty. The second one is for the author year scheme. When you use model2-names.bst, the citation commands will be like `\citep`, `\citet`, `\citealt` etc. However when you use model1-num-names.bst, you may use only `\cite` command.

`thebibliography` environment. Each reference is a `\bibitem` and each `\bibitem` is identified by a label, by which it can be cited in the text:
In connection with cross-referencing and possible future hyperlinking it is not a good idea to collect more that one literature item in one `\bibitem`. The so-called Harvard or author-year style of referencing is enabled by the LaTeX package natbib. With this package the literature can be cited as follows:

- Parenthetical: `\citep{WB96}` produces (Wettig & Brown, 1996).
- Textual: `\citet{ESG96}` produces Elson et al. (1996).
- An affix and part of a reference: `\citep[e.g.][Ch. 2]{Gea97}` produces (e.g. Governato et al., 1997, Ch. 2).

In the numbered scheme of citation, `\cite{<label>}` is used, since `\citep` or `\citet` has no relevance in the numbered scheme. natbib package is loaded by cas-sc with `numbers` as default option. You can change this to author-year or harvard scheme by adding option `authoryear` in the class loading command. If you want to use more options of the natbib package, you can do so with the `\biboptions` command. For details of various options of the natbib package, please take a look at the natbib documentation, which is part of any standard LaTeX installation.

A. My Appendix

Appendix sections are coded under `\appendix`.

`\printcredits` command is used after appendix sections to list author credit taxonomy contribution roles tagged using `\credit` in frontmatter.

CRediT authorship contribution statement

CV Radhakrishnan: Conceptualization of this study, Methodology, Software. **CV Rajagopal:** Data curation, Writing - Original draft preparation.

References

Fortunato, S., 2010. Community detection in graphs. Phys. Rep.-Rev. Sec. Phys. Lett. 486, 75–174.

Newman, M.E.J., Girvan, M., 2004. Finding and evaluating community structure in networks. Phys. Rev. E. 69, 026113.

Vehlow, C., Reinhardt, T., Weiskopf, D., 2013. Visualizing fuzzy overlapping communities in networks. IEEE Trans. Vis. Comput. Graph. 19, 2486–2495.

Author biography without author photo. Author biography.

Author biography with author photo. Author biography.

Author biography with author photo. Author biography. Author biography. Author biography. Author biography. Author biography. Author biography. Author biography. Author biography. Author biography. Author biography. Author biography. Author biography. Author biography.
