# Supplementary material for: RAP3DF - One shoot 3D face dataset
Source: Data Brief. 2020 Sep 5;32:106281. doi: 10.1016/j.dib.2020.106281 (PMC7509182; doi:10.1016/j.dib.2020.106281)
Supplement: Supplementary file 1 [file mmc1.zip › doc/elsdoc-cas.pdf]

Documentation for Elsevier's Complex Article Service (CAS) LaTeX template

Elsevier Ltd

QUICK LINKS

- ► Introduction
- ► Front matter
- ► Main Matter
- ► CRediT...
- ► Bibliography

1. Introduction

Two classfiles namely cas-sc.cls and cas-dc.cls were written for typesetting articles submitted in journals of Elsevier's Complex Article Service (CAS) workflow.

1.1. Usage

1. cas-sc.cls for single column journals.

```
\documentclass[<options>]{cas-sc}
```

2. cas-dc.cls for single column journals.

```
\documentclass[<options>]{cas-dc}
```

and have an option longmktitle to handle long front matter.

2. Front matter

```
\title [mode = title]{This is a specimen $a_b$ title}
\tnotemark[1,2]

\tnotetext[1]{This document is the results of the research
  project funded by the National Science Foundation.}

\tnotetext[2]{The second title footnote which is a longer text
  matter to fill through the whole text width and overflow into
  another line in the footnotes area of the first page.}

\author[1,3]{CV Radhakrishnan}[type=editor,
                auid=000,bioid=1,
                prefix=Sir,
                role=Researcher,
                orcid=0000-0001-7511-2910]
\cormark[1]
\fnmark[1]
\ead{cvr_1@tug.org.in}
\ead[url]{www.cvr.cc, cvr@sayahna.org}
```

© 2019, Elsevier Ltd. Bugs, feature requests, suggestions and comments shall be mailed to <elsarticle@stmdocs.in>.

Version: 1.0
Date: February 11, 2019
Contact: elsarticle@stmdocs.in

```
\credit{Conceptualization of this study, Methodology,
     Software}

\address[1]{Elsevier B.V., Radarweg 29, 1043 NX Amsterdam,
        The Netherlands}

\author[2,4]{Han Theh Thanh}[style=chinese]

\author[2,3]{CV Rajagopal}[%
  role=Co-ordinator,
  suffix=Jr,
  ]
\fnmark[2]
\ead{cvr3@sayahna.org}
\ead[URL]{www.sayahna.org}

\credit{Data curation, Writing - Original draft preparation}

\address[2]{Sayahna Foundation, Jagathy, Trivandrum 695014,
        India}

\author[1,3]{Rishi T.}
\cormark[2]
\fnmark[1,3]
\ead{rishi@stmdocs.in}
\ead[URL]{www.stmdocs.in}

\address[3]{STM Document Engineering Pvt Ltd., Mepukada,
   Malayinkil, Trivandrum 695571, India}

\cortext[cor1]{Corresponding author}
\cortext[cor2]{Principal corresponding author}
\fntext[fn1]{This is the first author footnote. but is common
  to third author as well.}
\fntext[fn2]{Another author footnote, this is a very long
  footnote and it should be a really long footnote. But this
  footnote is not yet sufficiently long enough to make two lines
  of footnote text.}
```

```
\nonumnote{This note has no numbers. In this work we
  demonstrate $a_b$ the formation Y\_1 of a new type of
  polariton on the interface between a cuprous oxide slab
  and a polystyrene micro-sphere placed on the slab.
  }

\begin{abstract}[S U M M A R Y]
This template helps you to create a properly formatted
 \LaTeX\ manuscript.

\noindent\texttt{\textbackslash begin{abstract}} \dots
\texttt{\textbackslash end{abstract}} and
\verb+\begin{keyword}+ \verb+...+ \verb+\end{keyword}+
which contain the abstract and keywords respectively.
Each keyword shall be separated by a \verb+\sep+ command.
\end{abstract}

\begin{keywords}
quadrupole exciton \sep polariton \sep \WGM \sep \BEC
\end{keywords}

\maketitle
```

2.1. Title

`\title` command have the below options:

1. `title`: Document title
2. `alt`: Alternate title
3. `sub`: Sub title
4. `trans`: Translated title
5. `transsub`: Translated sub title

```
\title[mode=title]{This is a title}
\title[mode=alt]{This is a alternate title}
\title[mode=sub]{This is a sub title}
\title[mode=trans]{This is a translated title}
\title[mode=transsub]{This is a translated sub title}
```

This is a specimen a_b title[⋆,⋆⋆]

Sir CV Radhakrishnan[a,c,∗,1] (Researcher), Han Theh Thanh[b,d], CV Rajagopal Jr[b,c,2] (Co-ordinator) and Rishi T.[a,c,∗∗,1,3]

[a]*Elsevier B.V., Radarweg 29, 1043 NX Amsterdam, The Netherlands*
[b]*Sayahna Foundation, Jagathy, Trivandrum 695014, India*
[c]*STM Document Engineering Pvt Ltd., Mepukada, Malayinkil, Trivandrum 695571, India*

ARTICLE INFO

Keywords:
quadrupole exciton
polariton
WGM
BEC

ABSTRACT

This template helps you to create a properly formatted LaTeX manuscript. `\beginabstract` ... `\endabstract` and `\begin{keyword}` ... `\end{keyword}` which contain the abstract and keywords respectively. Each keyword shall be separated by a `\sep` command.

1. Introduction

The Elsevier cas-sc class is based on the standard article class and supports almost all of the functionality of that class. In addition, it features commands and options to format the

- document style
- baselineskip
- front matter
- keywords and MSC codes
- theorems, definitions and proofs
- lables of enumerations
- citation style and labeling.

This class depends on the following packages for its proper functioning:

1. natbib.sty for citation processing;
2. geometry.sty for margin settings;
3. fleqn.clo for left aligned equations;
4. graphicx.sty for graphics inclusion;
5. hyperref.sty optional packages if hyperlinking is required in the document;

All the above packages are part of any standard LaTeX installation. Therefore, the users need not be bothered about downloading any extra packages.

[⋆] This document is the results of the research project funded by the National Science Foundation.
[⋆⋆] The second title footnote which is a longer text matter to fill through the whole text width and overflow into another line in the footnotes area of the first page.

This note has no numbers. In this work we demonstrate a_b the formation Y_1 of a new type of polariton on the interface between a cuprous oxide slab and a polystyrene micro-sphere placed on the slab.

[∗] Corresponding author
[∗∗] Principal corresponding author
cvr_1@tug.org.in (C. Radhakrishnan); cvr3@sayahna.org (C. Rajagopal); rishi@stmdocs.in (R. T.)
www.cvr.cc, cvr@sayahna.org (C. Radhakrishnan); www.sayahna.org (C. Rajagopal); www.stmdocs.in (R. T.)
ORCID(s): 0000-0001-7511-2910 (C. Radhakrishnan)

[1] This is the first author footnote. but is common to third author as well.
[2] Another author footnote, this is a very long footnote and it should be a really long footnote. But this footnote is not yet sufficiently long enough to make two lines of footnote text.

Rawat et al.: *Preprint submitted to Elsevier* Page 1 of 5

Clip 1: Single column output (classfile: cas-sc.cls).

This is a specimen a_b title[⋆,⋆⋆]

Sir CV Radhakrishnan[a,c,∗,1] (Researcher), Han Theh Thanh[b,d], CV Rajagopal Jr[b,c,2] (Co-ordinator) and Rishi T.[a,c,∗∗,1,3]

[a]Elsevier B.V., Radarweg 29, 1043 NX Amsterdam, The Netherlands
[b]Sayahna Foundation, Jagathy, Trivandrum 695014, India
[c]STM Document Engineering Pvt Ltd., Mepukada, Malayinkil, Trivandrum 695571, India

ARTICLE INFO

Keywords:
quadrupole exciton
polariton
WGM
BEC

ABSTRACT

This template helps you to create a properly formatted LaTeX manuscript.
`\begin{abstract}` ... `\end{abstract}` and `\begin{keyword}` ... `\end{keyword}` which contain the abstract and keywords respectively. Each keyword shall be separated by a `\sep` command.

1. Introduction

The Elsevier cas-dc class is based on the standard article class and supports almost all of the functionality of that class. In addition, it features commands and options to format the

- document style
- baselineskip
- front matter
- keywords and MSC codes
- theorems, definitions and proofs
- lables of enumerations
- citation style and labeling.

This class depends on the following packages for its proper functioning:

1. natbib.sty for citation processing;
2. geometry.sty for margin settings;
3. fleqn.clo for left aligned equations;
4. graphicx.sty for graphics inclusion;
5. hyperref.sty optional packages if hyperlinking is required in the document;

All the above packages are part of any standard LaTeX installation. Therefore, the users need not be bothered about downloading any extra packages.

2. Installation

The package is available at author resources page at Elsevier (`http://www.elsevier.com/locate/latex`). The class may be moved or copied to a place, usually, `$TEXMF/tex/latex/elsevier/`, or a folder which will be read by LaTeX during document compilation. The TeX file database needs updation after moving/copying class file. Usually, we use commands like `mktexlsr` or `texhash` depending upon the distribution and operating system.

3. Front matter

The author names and affiliations could be formatted in two ways:

(1) Group the authors per affiliation.
(2) Use footnotes to indicate the affiliations.

See the front matter of this document for examples. You are recommended to conform your choice to the journal you are submitting to.

4. Bibliography styles

There are various bibliography styles available. You can select the style of your choice in the preamble of this document. These styles are Elsevier styles based on standard styles like Harvard and Vancouver. Please use BibTeX to generate your bibliography and include DOIs whenever available.

Here are two sample references: [7] [7, 8] [7, 9]

5. Floats

Figures may be included using the command, `\includegraphics` in combination with or without its several options to further control graphic. `\includegraphics`

[⋆] This document is the results of the research project funded by the National Science Foundation.

[⋆⋆] The second title footnote which is a longer text matter to fill through the whole text width and overflow into another line in the footnotes area of the first page.

This note has no numbers. In this work we demonstrate a_b the formation Y_1 of a new type of polariton on the interface between a cuprous oxide slab and a polystyrene micro-sphere placed on the slab.

[∗] Corresponding author
[∗∗] Principal corresponding author
cvr_1@tug.org.in (C. Radhakrishnan); cvr3@sayahna.org (C. Rajagopal); rishi@stmdocs.in (R. T.)
www.cvr.cc, cvr@sayahna.org (C. Radhakrishnan); www.sayahna.org (C. Rajagopal); www.stmdocs.in (R. T.)
ORCID(s): 0000-0001-7511-2910 (C. Radhakrishnan)

[1] This is the first author footnote. but is common to third author as well.

[2] Another author footnote, this is a very long footnote and it should be a really long footnote. But this footnote is not yet sufficiently long enough to make two lines of footnote text.

Rawat et al.: *Preprint submitted to Elsevier* Page 1 of 3

Clip 2: Double column output (classfile: cas-dc.cls).

2.2. Author

`\author` command have the below options:

1. `auid:` Author id
2. `bioid:` Biography id
3. `alt:` Alternate author
4. `style:` Style of author name chinese
5. `prefix:` Prefix Sir
6. `suffix:` Suffix
7. `degree:` Degree
8. `role:` Role
9. `orcid:` ORCID
10. `collab:` Collaboration
11. `anon:` Anonymous author
12. `deceased:` Deceased author
13. `twitter:` Twitter account
14. `facebook:` Facebook account
15. `linkedin:` LinkedIn account
16. `plus:` Google plus account
17. `gplus:` Google plus account

```
\author[1,3]{Author Name}[type=editor,
   auid=000,bioid=1,
   prefix=Sir,
   role=Researcher,
   orcid=0000-0001-7511-2910,
   facebook=<facebook id>,
   twitter=<twitter id>,
   linkedin=<linkedin id>,
   gplus=<gplus id>]
```

© 2019, Elsevier Ltd. Bugs, feature requests, suggestions and comments shall be mailed to <elsarticle@stmdocs.in>.

2.3. Various Marks in the Front Matter

The front matter becomes complicated due to various kinds of notes and marks to the title and author names. Marks in the title will be denoted by a star (⋆) mark; footnotes are denoted by super scripted Arabic numerals, corresponding author by of an Conformal asterisk (*) mark.

2.3.1. Title marks

Title mark can be entered by the command, \tnotemark[<num>] and the corresponding text can be entered with the command \tnotetext[<num>]{<text>}. An example will be:

```
\title[mode=title]{Leveraging social media news to predict
               stock index movement using RNN-boost}

\tnotemark[1,2]

\tnotetext[1]{This document is the results of the research
  project funded by the National Science Foundation.}

\tnotetext[2]{The second title footnote which is a longer
  text matter to fill through the whole text width and
  overflow into another line in the footnotes area of
  the first page.}
```

\tnotetext and \tnotemark can be anywhere in the front matter, but shall be before \maketitle command.

2.3.2. Author marks

Author names can have many kinds of marks and notes:

```
    footnote mark : \fnmark[<num>]
    footnote text : \fntext[<num>]{<text>}
    affiliation mark : \author[<num>]
    email : \ead{<emailid>}
    url : \ead[url]{<url>}
    corresponding author mark : \cormark[<num>]
    corresponding author text : \cortext[<num>]{<text>}
```

2.3.3. Other marks

At times, authors want footnotes which leave no marks in the author names. The note text shall be listed as part of the front matter notes. Class files provides \nonumnote for this purpose. The usage

© 2019, Elsevier Ltd. Bugs, feature requests, suggestions and comments shall be mailed to <elsarticle@stmdocs.in>.

```
\nonumnote{<text>}
```

and should be entered anywhere before the \maketitle command for this to take effect.

2.4. Abstract and Keywords

Abstract shall be entered in an environment that starts with \begin{abstract} and ends with \end{abstract}. Longer abstracts spanning more than one page is also possible in Class file even in double column mode. We need to invoke longmktitle option in the class loading line for this to happen smoothly.

The key words are enclosed in a {keyword} environment.

```
\begin{abstract}
 This is a abstract. \lipsum[3]
\end{abstract}

\begin{keywords}
 First keyword \sep Second keyword \sep Third
   keyword \sep Fourth keyword
\end{keywords}
```

3. Main Matter

3.1. Tables

3.1.1. Normal tables

```
\begin{table}
  \caption{This is a test caption.}
  \begin{tabular*}{\tblwidth}{@{} LLLL@{} }
   \toprule
    Col 1 & Col 2\\
   \midrule
    12345 & 12345\\
    12345 & 12345\\
    12345 & 12345\\
   \bottomrule
  \end{tabular*}
\end{table}
```

3.1.2. Span tables

```
\begin{table*}[width=.9\textwidth,cols=4,pos=h]
 \caption{This is a test caption.}
 \begin{tabular*}{\tblwidth}{@{} LLLLLL@{} }
  \toprule
   Col 1 & Col 2 & Col 3 & Col4 & Col5 & Col6 & Col7\\
  \midrule
   12345 & 12345 & 123 & 12345 & 123 & 12345 & 123 \\
   12345 & 12345 & 123 & 12345 & 123 & 12345 & 123 \\
   12345 & 12345 & 123 & 12345 & 123 & 12345 & 123 \\
  \bottomrule
 \end{tabular*}
\end{table*}
```

3.2. Figures

3.2.1. Normal figures

```
\begin{figure}
\centering
\includegraphics[scale=.75]{Fig1.pdf}
\caption{The evanescent light - $1S$ quadrupole coupling
($g_{1,l}$) scaled to the bulk exciton-photon coupling
($g_{1,2}$). The size parameter $kr_{0}$ is denoted as $x$ and
the \PMS is placed directly on the cuprous oxide sample ($\delta
r=0$, See also Fig. \protect\ref{FIG:2}).}
\label{FIG:1}
\end{figure}
```

3.2.2. Span figures

```
\begin{figure*}
\centering
 \includegraphics[width=\textwidth,height=2in]{Fig2.pdf}
\caption{Schematic of formation of the evanescent polariton on
linear chain of \PMS. The actual dispersion is determined by
 the ratio of two coupling parameters such as exciton-\WGM
 coupling and \WGM-\WGM coupling between the microspheres.}
 \label{FIG:2}
\end{figure*}
```

3.3. Theorem and theorem like environments

CAS class file provides a few hooks to format theorems and theorem like environments with ease. All commands the options that are used with `\newtheorem` command will work exactly in the same manner. Class file provides three commands to format theorem or theorem like environments:

1. `\newtheorem` command formats a theorem in LaTeX's default style with italicized font for theorem statement, bold weight for theorem heading and theorem number typeset at the right of theorem heading. It also optionally accepts an argument which will be printed as an extra heading in parentheses. Here is an example coding and output:

```
\newtheorem{theorem}{Theorem}
\begin{theorem}\label{thm}
 The \WGM evanescent field penetration depth into the
 cuprous oxide adjacent crystal is much larger than the
 \QE radius:
 \begin{equation*}
  \lambda_{1S}/2 \pi \left({\epsilon_{Cu2O}-1}
    \right)^{1/2} = 414 \mbox{ \AA} \gg a_B = 4.6
    \mbox{ \AA}
 \end{equation*}
\end{theorem}
```

2. `\newdefinition` command does exactly the same thing as with except that the body font is up-shape instead of italic. See the example below:

```
\newdefinition{definition}{Definition}
\begin{definition}
 The bulk and evanescent polaritons in cuprous oxide
 are formed through the quadrupole part of the light-matter
 interaction:
 \begin{equation*}
  H_{int} = \frac{i e }{m \omega_{1S}} {\bf E}_{i,s}
    \cdot {\bf p}
 \end{equation*}
\end{definition}
```

© 2019, Elsevier Ltd. Bugs, feature requests, suggestions and comments shall be mailed to <elsarticle@stmdocs.in>.

3. \newproof command helps to define proof and custom proof environments without counters as provided in the example code. Given below is an example of proof of theorem kind.

```
\newproof{pot}{Proof of Theorem \ref{thm}}
\begin{pot}
 The photon part of the polariton trapped inside the \PMS
 moves as it would move in a micro-cavity of the effective
 modal volume $V \ll 4 \pi r_{0}^{3} /3$. Consequently, it
 can escape through the evanescent field. This evanescent
 field essentially has a quantum origin and is due to
 tunneling through the potential caused by dielectric
 mismatch on the \PMS surface. Therefore, we define the
 \emph{evanescent} polariton (\EP) as an evanescent light -
 \QE coherent superposition.
\end{pot}
```

3.4. Enumerated and Itemized Lists

CAS class files provides an extended list processing macros which makes the usage a bit more user friendly than the default LaTeX list macros. With an optional argument to the \begin{enumerate} command, you can change the list counter type and its attributes. You can see the coding and typeset copy.

```
\begin{enumerate}[1.]
 \item The enumerate environment starts with an optional
     argument `1.' so that the item counter will be suffixed
     by a period as in the optional argument.
 \item If you provide a closing parenthesis to the number in the
     optional argument, the output will have closing
     parenthesis for all the item counters.
 \item You can use `(a)' for alphabetical counter and `(i)' for
     roman counter.
 \begin{enumerate}[a)]
  \item Another level of list with alphabetical counter.
  \item One more item before we start another.
  \begin{enumerate}[(i)]
   \item This item has roman numeral counter.
```

```
    \item Another one before we close the third level.
   \end{enumerate}
   \item Third item in second level.
  \end{enumerate}
  \item All list items conclude with this step.
\end{enumerate}

\section{Biography}

\verb+\bio+ command have the below options:
\begin{enumerate}
 \item \verb+width:+ Width of the author photo (default is 1in).
 \item \verb+pos:+ Position of author photo.
\end{enumerate}

\begin{vquote}
\bio[width=10mm,pos=l]{tuglogo.jpg}
 \textbf{Another Biography:}
  Recent experimental \cite{HARA:2005} and theoretical
  \cite{DEYCH:2006} studies have shown that the \WGM can travel
  along the chain as "heavy photons". Therefore the \WGM
  acquires the spatial dispersion, and the evanescent
  quadrupole polariton has the form (See Fig.\ref{FIG:3}):
\endbio
```

4. CRediT authorship contribution statement

Give the authorship contribution after each author as

```
\credit{Conceptualization of this study, Methodology,
     Software}
```

To print the details use `\printcredits`

```
\author[1,3]{V. {{\=A}}nand Rawat}[auid=000,
            bioid=1,
            prefix=Sir,
            role=Researcher,
            orcid=0000-0001-7511-2910]
```

```
\cormark[1]
\fnmark[1]
\ead{cvr_1@tug.org.in}
\ead[url]{www.cvr.cc, www.tug.org.in}

\credit{Conceptualization of this study, Methodology,
      Software}

\address[1]{Indian \TeX{} Users Group, Trivandrum 695014,
    India}

\author[2,4]{Han Theh Thanh}[style=chinese]

\author[2,3]{T. Rishi Nair}[role=Co-ordinator,
            suffix=Jr]
\fnmark[2]
\ead{rishi@sayahna.org}
\ead[URL]{www.sayahna.org}

\credit{Data curation, Writing - Original draft preparation}

. . .
. . .
. . .
\printcredits
```

5. Bibliography

For CAS categories, two reference models are recommended. They are model1-num-names.bst and model2-names.bst. Former will format the reference list and their citations according to numbered scheme whereas the latter will format according name-date or author-year style. Authors are requested to choose any one of these according to the journal style. You may download these from

The above bsts are available in the following location for you to download:

https://support.stmdocs.in/wiki/index.php?title=Model-wise_bibliographic_style_files □
