# Supplementary figures and images for: RAP3DF - One shoot 3D face dataset
Source: Data Brief. 2020 Sep 5;32:106281. doi: 10.1016/j.dib.2020.106281 (PMC7509182; doi:10.1016/j.dib.2020.106281)

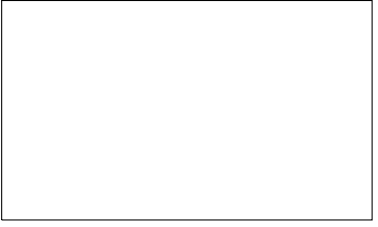

Supplement: Supplementary file 1 [file mmc1.zip › figs/grabs.pdf]

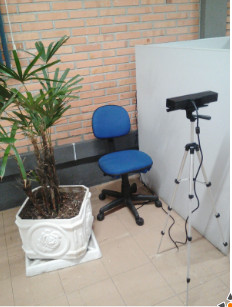

Supplement: Supplementary file 1 [file mmc1.zip › figs/estudio_01.jpg]

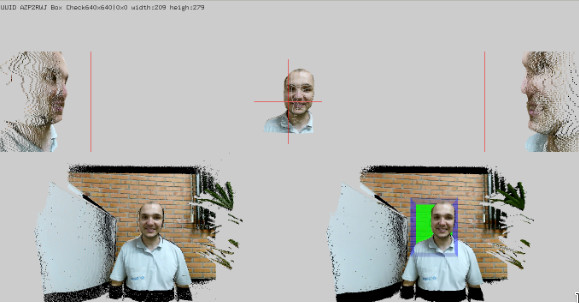

Supplement: Supplementary file 1 [file mmc1.zip › figs/estudio_02.jpg]

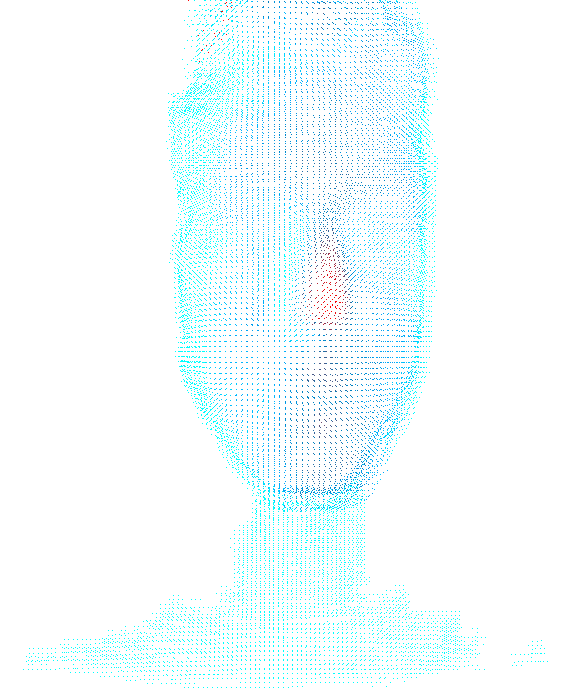

Supplement: Supplementary file 1 [file mmc1.zip › figs/pontos_f.png]

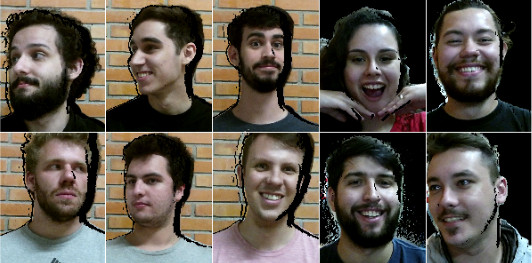

Supplement: Supplementary file 1 [file mmc1.zip › figs/resultado_imagens_aleatorias.jpg]

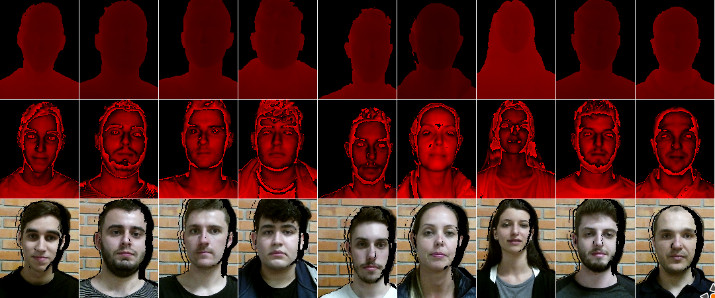

Supplement: Supplementary file 1 [file mmc1.zip › figs/resultado_imagens_faciais.jpg]

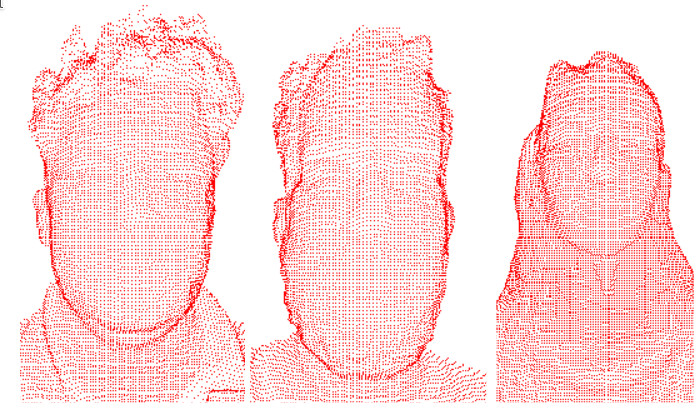

Supplement: Supplementary file 1 [file mmc1.zip › figs/resultado_imagens_pontos.jpg]

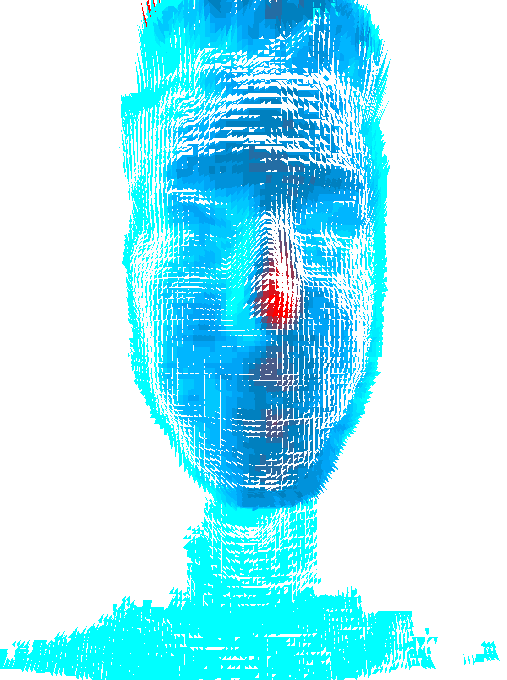

Supplement: Supplementary file 1 [file mmc1.zip › figs/triang_f.png]

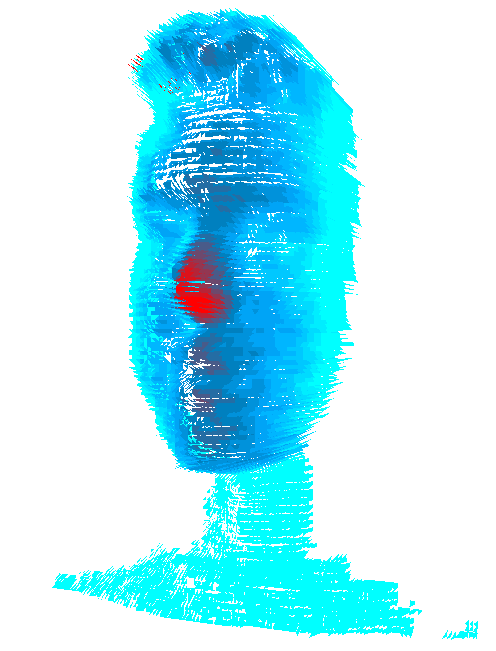

Supplement: Supplementary file 1 [file mmc1.zip › figs/triang_lf.png]

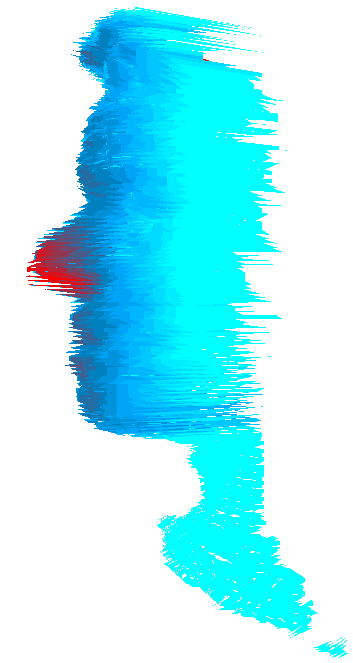

Supplement: Supplementary file 1 [file mmc1.zip › figs/triang_ll.png]

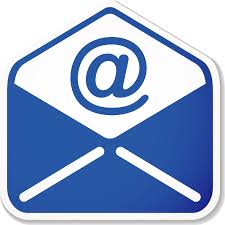

Supplement: Supplementary file 1 [file mmc1.zip › thumbnails/cas-email.jpeg]

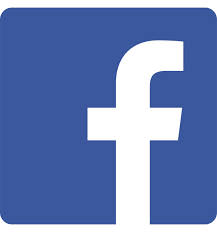

Supplement: Supplementary file 1 [file mmc1.zip › thumbnails/cas-facebook.jpeg]

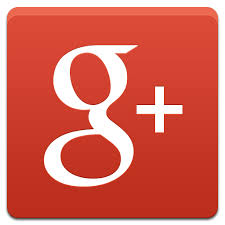

Supplement: Supplementary file 1 [file mmc1.zip › thumbnails/cas-gplus.jpeg]

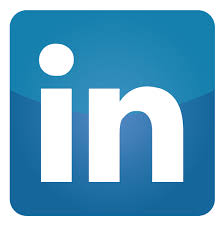

Supplement: Supplementary file 1 [file mmc1.zip › thumbnails/cas-linkedin.jpeg]

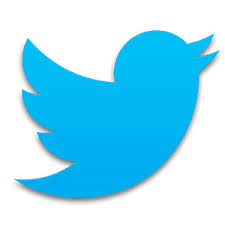

Supplement: Supplementary file 1 [file mmc1.zip › thumbnails/cas-twitter.jpeg]

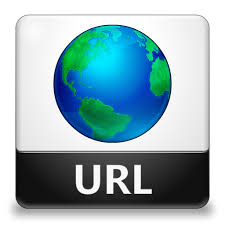

Supplement: Supplementary file 1 [file mmc1.zip › thumbnails/cas-url.jpeg]
